# Construction of a prognostic model for disulfidptosis related ferroptosis genes lung adenocarcinoma and the role of DECR1 in lung adenocarcinoma

**DOI:** 10.3389/fimmu.2025.1658897

**Published:** 2025-10-15

**Authors:** Haoran Liu, Ying Zhang, Yuqing Dong, Xintong Jiang, Shuyang Xie, Pingyu Wang

**Affiliations:** ^1^ School of Public Health, Binzhou Medical University, Yantai, Shandong, China; ^2^ Department of Biochemistry and Molecular Biology, Binzhou Medical University, Yantai, Shandong, China; ^3^ Shandong Laboratory of Advanced Materials and Green Manufacturing, Binzhou Medical University, Yantai, Shandong, China

**Keywords:** lung adenocarcinoma, disulfidptosis, prognostic mode, ferroptosis, biomarker

## Abstract

**Background:**

Lung adenocarcinoma (LUAD) is one of the common malignant tumors worldwide, and the 5-year survival rate remains unsatisfactory. To investigate the association between disulfidptosis-related ferroptosis genes (DFRGs) and the prognosis of patients with LUAD, establish a risk prognostic model, validate key biomarkers *in vitro*, and provide references for the prognosis of LUAD patients.

**Methods:**

R software was employed to identify DFRGs. Univariate Cox regression and Lasso-Cox regression analyses were combined to construct a risk score prognostic model. The predictive power of the model was evaluated using Kaplan-Meier survival curves, receiver operating characteristic (ROC) curves, and calibration curves. Immune-related functions, tumor mutation burden, and single-cell analyses were performed on the model genes. Finally, *in vitro* validation of key prognostic markers was conducted via qRT-PCR, wound healing assay, Transwell assay, CCK8 assay, and flow cytometry apoptosis assay.

**Results:**

Six DFRGs were screened through univariate Cox regression and Lasso-Cox regression analyses to construct the prognostic model. The areas under the ROC curve (AUC) for 1, 2, and 3 years in the training set were 0.836, 0.771, and 0.786, respectively. Decision curve analysis (DCA) indicated that the risk score model effectively predicted lung adenocarcinoma prognosis. *In vitro* validation demonstrated that knockdown of DECR1 significantly suppressed lung adenocarcinoma cell proliferation and migration, and promoted cell apoptosis (*P* < 0.05).

**Conclusion:**

This study established a risk score model based on six DFRGs, which demonstrated favorable prognostic value. DECR1 promotes the progression of LUAD and holds promise as an effective biomarker.

## 1 Introduction

Lung cancer is one of the most common malignant tumors and is highly lethal, being the leading cause of cancer-related deaths worldwide. Due to the extremely limited treatment options, the survival rate for patients with advanced disease is very low (1). It is expected that by 2035, the incidence of lung cancer will further increase in most countries, making it the main challenge for global public health issues. According to data from the National Cancer Center, lung cancer is the leading cause of cancer in China, with 710,000 deaths, accounting for 23.8% of all cancer deaths, of which lung adenocarcinoma (LUAD) has the highest proportion (2), and has become a key focus in clinical and translational research.

The human battle against lung cancer is a journey marked by the continuous evolution of treatment strategies. In the early days, treatment centered on traditional chemotherapy; these drugs inhibit tumor cell proliferation by damaging DNA, but they lack specificity, often causing severe side effects and leading to rapid tumor drug resistance (3). Later, the emergence of targeted therapies, such as EGFR inhibitors, enabled precise targeting of tumors. However, these therapies are prone to drug resistance, and studies have found that approximately 15.84% of patients with EGFR-TKI resistance develop MET amplification (4). In recent years, immune checkpoint inhibitors, such as PD-1 antibodies, have revolutionized the treatment landscape. Nevertheless, they still have limitations, including a relatively low overall response rate, immune-related adverse reactions, and the risk of drug resistance (5). Additionally, unresolved issues persist, such as the inability of traditional staging methods to accurately predict prognosis and the high rate of metastasis or recurrence after treatment. Against this backdrop, exploring new mechanisms of tumor cell death has become a key breakthrough. The traditional apoptotic pathway is less effective due to tumor drug resistance, while non-apoptotic regulated cell death pathways, such as ferroptosis and disulfidptosis, which can bypass drug resistance and exhibit high selectivity for tumor cells, thus offering a new direction for lung cancer treatment.

Disulfidptosis is a recently discovered type of cell death within the biological realm. It occurs when the levels of the solute carrier family 7 member 11 (SLC7A11) gene rise in cells facing a shortage of glucose (6). The regulation of disulfidptosis involves the formation and breaking of disulfide bonds, as well as the involvement of proteins such as NCKAP1 and signaling pathways related to redox and cellular metabolism. Disulfidptosis has the potential to serve as a target for cancer treatment. Ferroptosis is another mode of cell death that is iron-dependent and plays a critical role in tumorigenesis and tumor progression. During the development of tumors, ferroptosis has a dual role, both promoting and inhibiting tumor growth (7). Additionally, it can markedly influence the effectiveness of chemotherapy, radiotherapy, and immunotherapy in cancer patients (8). Ferroptosis plays a crucial role in cancer treatment. Compared to normal cells, tumor cells usually contain higher levels of iron and are more dependent on it (9). Therefore, by inducing ferroptosis, cancer cells can be selectively killed while causing minimal damage to non-malignant cells.

Although disulfidptosis and ferroptosis are two distinct forms of regulated cell death, they share common regulatory factors. The expression of SLC7A11, a key regulatory gene in disulfidptosis, can influence intracellular iron levels (10). While studies on disulfidptosis-related genes and ferroptosis-related genes in LUAD exist separately, research combining both in the context of LUAD has not been reported. Therefore, this study innovatively links disulfidptosis and ferroptosis, aiming to fill the research gap on DFRGs in LUAD. Using public databases, we identified DFRGs, which can serve as molecular biomarkers for both disulfidptosis and ferroptosis. Subsequently, we constructed a DFRG-based prognostic model to predict the prognosis and immune scores of LUAD patients. This study is expected to provide decision-making guidance for personalized patient treatment.

## 2 Methods

### 2.1 LUAD data acquisition and preprocessing

The RNA sequencing data, along with clinical information and somatic mutation data of LUAD, were obtained from the TCGA database (https://portal.gdc.cancer.gov/repository) through the “TCGAbiolinks” R package, including data of 59 normal samples and 541 tumor samples. To convert the RNA sequencing data into transcripts per million (TPM) format, the “limma” package was employed, ensuring that the data from both sources were standardized. The copy number variation (CNV) of LUAD was also downloaded, and the CNV landscape was plotted. In addition, the datasets GSE30210 (n=226), GSE72094 (n=398), and GSE13213 (n=117) from the Gene Expression Omnibus (GEO) database (https://www.ncbi.nlm.nih.gov/geo/) were used as external validation datasets, and the GSE189357 dataset (n=9) was used for single-cell analysis.

### 2.2 Identification of DFRGs and differentially expressed genes

597 ferroptosis genes and 36 disulfidptosis genes were retrieved from the FerrDb database (www.zhounan.org/ferrdb/current) (11). The “limma” package in R software was utilized to perform Pearson correlation analysis on 597 ferroptosis genes and 36 disulfidptosis genes obtained from the FerrDb database, setting the correlation coefficient threshold at 0.3 to identify DFRGs. Subsequently, screen for differentially expressed genes between normal and tumor tissues in the TCGA-LUAD database, using an absolute log fold change (|logFC|) exceeding 2 and a false discovery rate (FDR) lower than 0.05 as the filtering criteria. Use the “ggplot2” and “ggrepel” packages in R software to create a volcano plot to display the results. Finally, take the intersection of the identified DFRGs and the differentially expressed genes in TCGA-LUAD to obtain the DFRGs in TCGA-LUAD, and use the “vnne” package in R software to create a Venn diagram for visualization (12).

### 2.3 Establishment and evaluation of the LUAD prognostic model

Divide the LUAD patients in the TCGA dataset into training and validation sets in a 6:4 ratio by using the “caret” R package. Establish a model related to DFRGs using the training set data and perform internal validation of the model’s accuracy and reliability using the validation set data. In the training set, identify DFRGs related to prognosis through univariate Cox regression analysis (13). To avoid overfitting, use the “glmnet” R package to perform LASSO regression analysis, selecting DFRGs with prognostic predictive value for multivariate Cox regression analysis. Ultimately, determine 6 DFRGs with independent prognostic value to construct a prognostic prediction model for LUAD, calculating the Risk score for each patient in the training sample. The calculation formula is: Risk score=∑Expi×coefi, where Expi and coefi represent the expression levels and coefficients of the prognostic DRGs, respectively. Subsequently, divide the LUAD patients into high-risk and low-risk groups based on the median value of the risk scores. Integrate the clinical data of the patients with the risk score data and perform survival analysis using the “survival” and “survminer” R packages, displaying the difference in overall survival (OS) between the high-risk and low-risk groups of patients using Kaplan-Meier (K-M) survival curves (14). Group patients based on different clinical characteristics to reveal differences in OS among different clinical subgroups and further validate the predictive efficacy of the model. Generate ROC curves using the “survminer” R package and the “timeROC” R package. Use the data set from the GEO database to conduct independent external validation analysis of the prognostic features.

### 2.4 Construction and assessment of the prognostic nomogram

Using univariate Cox regression analysis, we assessed the impact of risk scores and clinical characteristics on prognosis in TCGA-LUAD patients, and further incorporated statistically significant independent prognostic features into the multivariate Cox regression model to screen for factors that can independently predict prognosis. The results of the univariate and multivariate Cox regression analyses were visualized using forest plots generated with the “forestmodel” package. Additionally, the “rms” package was used to create C-index curves to evaluate the accuracy of the model’s predictive performance. A Nomogram is a visual representation that correlates various parameters on a single plane, reflecting the integration of multiple characteristics, and can predict individual patient survival rates. It has significant practical advantages in clinical settings. Based on age, gender, stage, and risk score, we constructed a Nomogram prediction model using univariate and multivariate Cox regression analyses with the “rms” package, predicting survival rates at 1, 2, and 3 years for LUAD patients. We also used calibration curves to assess the accuracy and reliability of the prediction model (15).

### 2.5 Immune landscape analysis of the prognostic model of DFRGs in LUAD

According to the analysis of immune-related functions, the differences in various immune functions between the high-risk and low-risk groups were demonstrated. The “estimate” package was used to evaluate the stromal cells, immune cells and comprehensive scores of LUAD patients, and the “ggpubr” package was employed to draw violin plots to assess the differences in Tumor Immune Microenvironment (TIME) scores among patients in the high-risk and low-risk groups. The TIDE scores of each LUAD patient were calculated through the TIDE website (http://tide.dfci.harvard.edu/), and the differences between the two groups were compared. Through the TIDE scores and TME scores, whether there were differences in the sensitivity to immunotherapy and the infiltration of the tumor microenvironment between the samples of the two groups was explored (12).

### 2.6 Tumor mutation burden analysis

Somatic mutation data for LUAD patients were downloaded from the TCGA database. Based on the total number of somatic mutations per megabase (Mb) of the exonic region in the human genome, we calculated the Tumor Mutation Burden (TMB) for each sample as the total number of somatic mutations divided by the effectively covered region. Visualization of somatic mutations in high-risk and low-risk groups was performed using the “maftools” R package, and the correlation between risk scores and TMB was computed. The “limma” and “survival” packages were employed to analyze differences in TMB between high- and low-risk groups and to assess the impact of TMB on the survival of LUAD patients (16).

### 2.7 Single-cell sequencing analysis

The study collected single-cell sequencing data for nine LUAD samples from the GEO database. The ScRNA-Seq data were processed by employing the “Seurat” R package, normalized and scaled using the “NormalizeData” and “ScaleData” functions, and batch effects mitigated by canonical correlation analysis. Cluster analysis was performed by using the t-distributed stochastic neighbor embedding (t-SNE) of “Seurat” along with the “FindClusters” function. Marker genes were delineated using the “FindAllMarkers” function in “Seurat” to select the cluster-specific marker (17).

### 2.8 Screening of potential therapeutic drugs for LUAD

Half inhibitory concentration (IC50) refers to the drug concentration when the antagonist can achieve 50% inhibition, which is generally used to indicate the ability of a drug or molecule to inhibit biological activities, such as cell activity rate. Therefore, the IC50 value can be used to measure the sensitivity of cells to different drugs, that is, the drug with higher IC50 value, the weaker the sensitivity of cells. Using the “oncoPredict” package, the Genomics of Drug Sensitivity in Cancer (GDSC) database was employed to forecast the treatment response to common anticancer drugs in the high and low risk groups, measure drug sensitivity according to the IC50 value, and evaluate the difference of the sensitivity of LUAD high and low risk groups to commonly used cancer treatments (18).

### 2.9 Cell culture and treatment

All the cell lines used in this study were purchased by the Shanghai Institute of Cell Biology, Chinese Academy of Sciences. We cultivated human normal lung epithelial cells BEAS-2B, along with lung adenocarcinoma cell line A549 and H1975, in 1640 medium supplemented with 10% fetal bovine serum, under conditions of 5% CO2 at a temperature of 37 °C. Cells in the logarithmic growth phase were utilized for the experiments (19).

Transfection reagent Liposomal lipofectin 2000 (Invitrogen, USA) was purchased from Invitrogene, and small interference RNA (siRNA) synthesis and negative control NC were purchased from Shanghai Jimma Pharmaceutical Technology Co., Ltd., and the procedures and systems were performed according to the manufacturer’s instructions. The sequences are as follows: for si-DECR1-1, the sense strand is GUGGAGAGGAAGUACUUAUTT, and the antisense strand is AUAAGUACUUCCUCUCCACTT; for si-DECR1-2, the sense strand is GCGAUUCAAUGUGAUUCAATT, and the antisense strand is UUGAAUCACAUUGAAUCGCTT. The siRNA negative control was purchased from Shanghai GenePharma Co., Ltd.

### 2.10 Quantitative real-time polymerase chain reaction

The PCR ARRAY was purchased from Shanghai Audubon Biotechnology Co., Ltd. (Shanghai, China). Total RNA was extracted by using Trizol reagent (Invitrogen, USA). Complementary DNA (cDNA) was synthesized using the PrimeScript RT kit (Takara) (20).

### 2.11 Wound healing assay

For wound-healing assays, cells were seeded using a 6-well plate. The cells were scratched using sterile tips perpendicular to previously labeled lines. After imaging the scratches with a light microscope, cell migration was measured at the time points of 0, 24, and 48h (21).

### 2.12 Transwell assay

The Transwell assays were performed using a 24-well transwell chamber to assess the mobility of the A549 and H1975 cell lines. Cells were seeded in serum-free medium, and the lower chamber was filled with 600µl of medium containing 30% serum. After 24h, the cells were fixed and stained (6).

### 2.13 CCK-8 assay

Cell proliferation was measured using the CCK 8 assay. A549 and H1975, cells were seeded in 96-well plates, and cultured for 0, 24, 48 and 72h, and CCK 8 solution was added and incubated for 1h in the dark. Absorbance values were measured at 450nm, using a microplate reader (22).

### 2.14 Flow cytometry

Cells were transfected for 24h and were cultured in serum-free medium for another 24h. Subsequently, the cells were harvested and stained using the Annexin and V-FITC Cell Apoptosis Detection Kit (Beyotime, Shanghai, China). Apoptosis was then determined by flow cytometry within 1h (23).

### 2.15 Statistical analysis

The Wilcoxon test was used to compare the two groups, and the Spearman correlation was employed to assess the correlation. All bioinformatics analyses were performed using R software (v.4.2.3) along with relevant packages and Perl 5.30.0 for statistical analysis. *P* < 0.05 was deemed statistically significant. *indicates *P* < 0.05, **indicates *P* < 0.01, ***indicates *P* < 0.001.

## 3 Results

### 3.1 Screening results of DFRGs in LUAD

**Figure 1:**
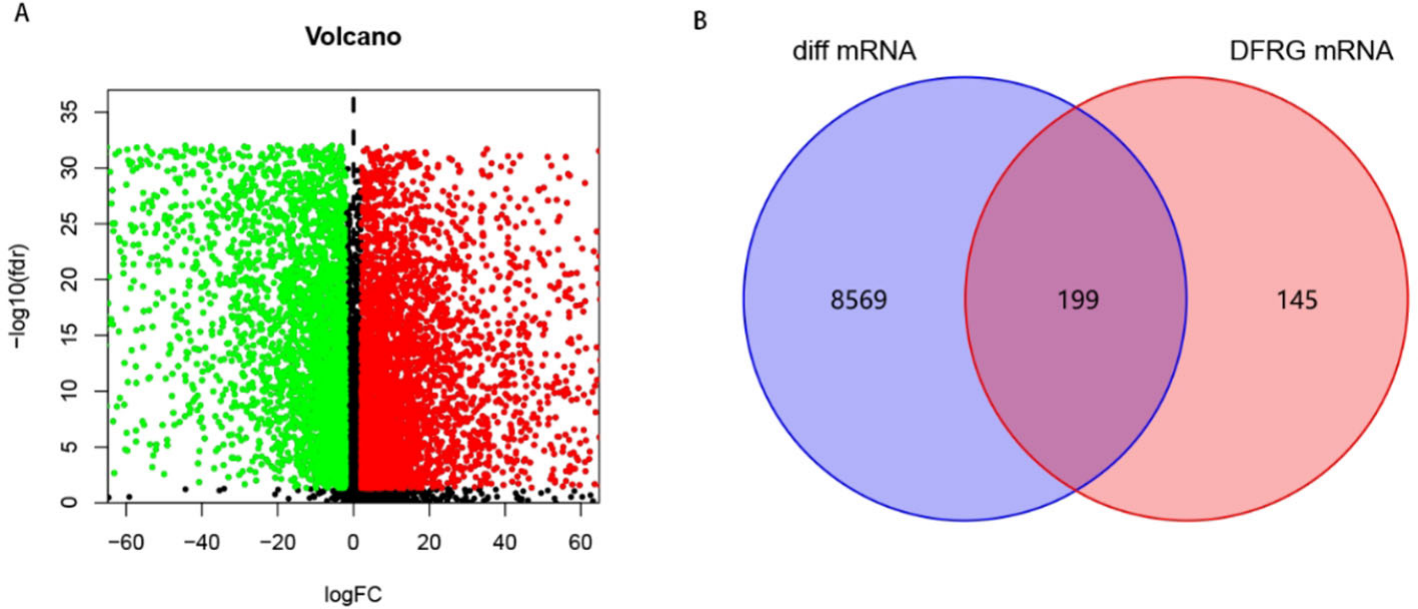
Screening of DFRGs. **(A)** Volcano plot of differential expression between normal and tumor in TCGA-LUAD. **(B)** Wayn diagram.

To explore DFRGs, 597 ferroptosis genes and 36 disulfidptosis genes downloaded from the FerrDb database were subjected for Pearson correlation analysis to yielded 344 DFRGs. TCGA-LUAD-RNA-seq data identified 19895 mRNAs that were differentially expressed between normal and tumor in total 8,768 using | logFC |> 2, FDR <0.05 as filtering criteria, and the results were visualized as volcano plots, with red and green dots indicating up-and downregulated mRNAs, respectively (**Figure 1A**). Finally, the filtered DFRGs were crossed with the differentially expressed mRNAs in TCGA-LUAD-RNA-seq data to obtain DFRGs in TCGA-LUAD-RNA-seq, and the results were visualized in Wayn diagram (**Figure 1B**). The 199 DFRGs were used for subsequent model-building studies.

### 3.2 Construction of DFRGs prognostic model

**Figure 2:**
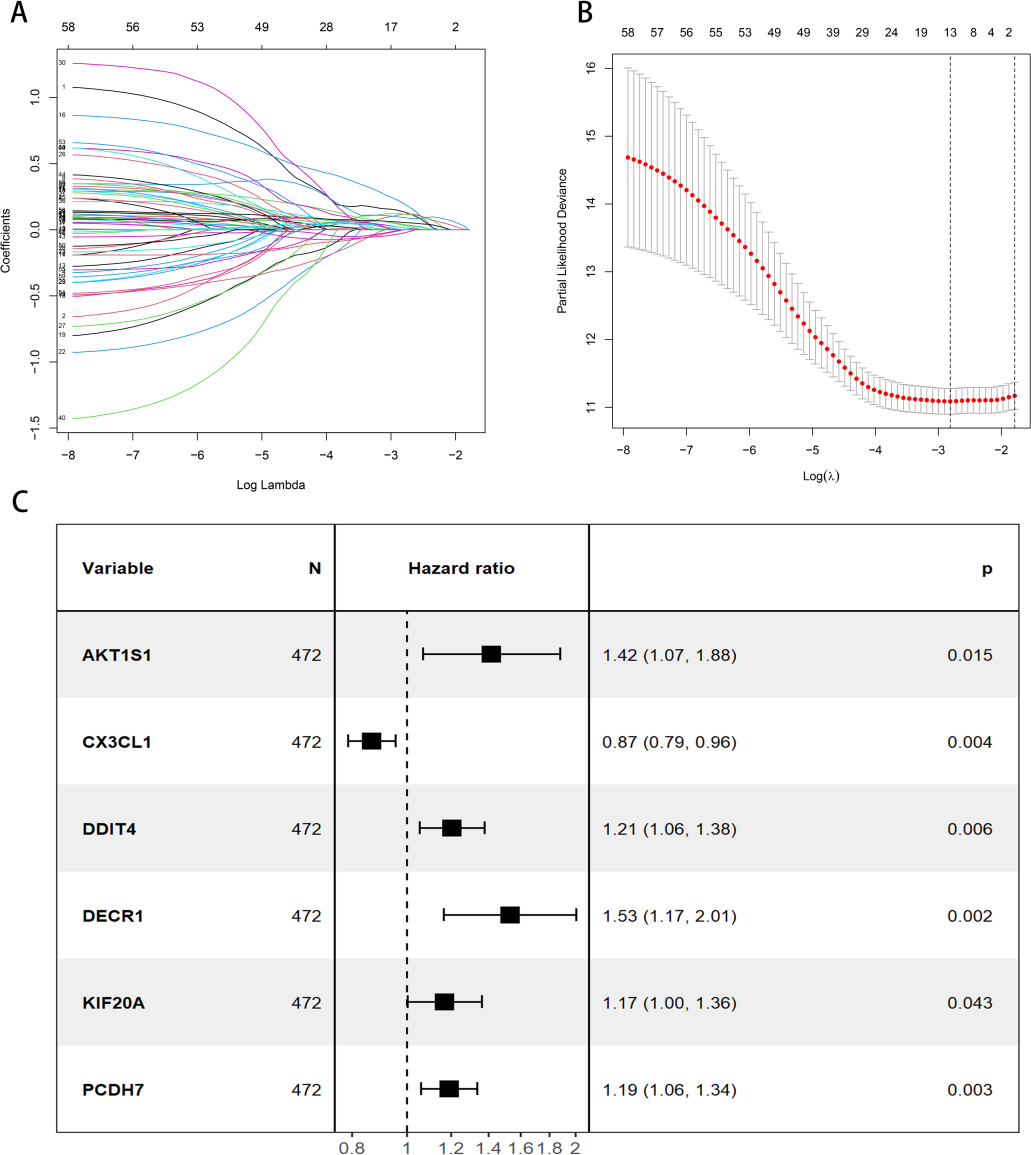
Construction of the prognostic model for DFRGs. **(A)** LASSO regression analysis. **(B)** λ selection diagram. **(C)** Forest plot of multivariate Cox regression.

A total of 472 patients were included except for samples with survival time < 30 days and missing clinical data. All the 199 DFRGs screened for genes associated with LUAD prognosis were *P* < 0.05, showing that 59 DFRGs were significantly associated with patient outcome. Subsequently, the above 59 DFRGs were further included in the Lasso regression analysis (**Figures 2A, B**) to obtain 13 meaningful DFRGs. Finally, the above 13 DFRGs were subjected to multivariate Cox regression analysis, resulting in 6 DFRGs for constructing the prognostic model. Among them, AKT1S1, DDIT 4, DECR1, KIF20A, and PCDH 7 (*HR* > 1) were risk factors, and CX3CL1 (*HR* < 1) were protective factors (**Figure 2C**). The hazard factor for each DFRGs was determined using Lasso-Cox regression, Patient risk scores were calculated based on the six DFRGs of the constructed model described above, The calculation formula is as follows: Riskscore = (0.4169×AKT1S1) + (-0.164×CX3CL1) + (0.2039×DDIT4) + (0.5426×DECR1) + (0.2968×KIF20A) + (0.1848×PCDH7). Patients in the training set, validation set, and total set were split into high risk and low risk categories based on their median patient risk score for later clinical value analysis.

### 3.3 Evaluation and validation of prognostic risk models

**Figure 3:**
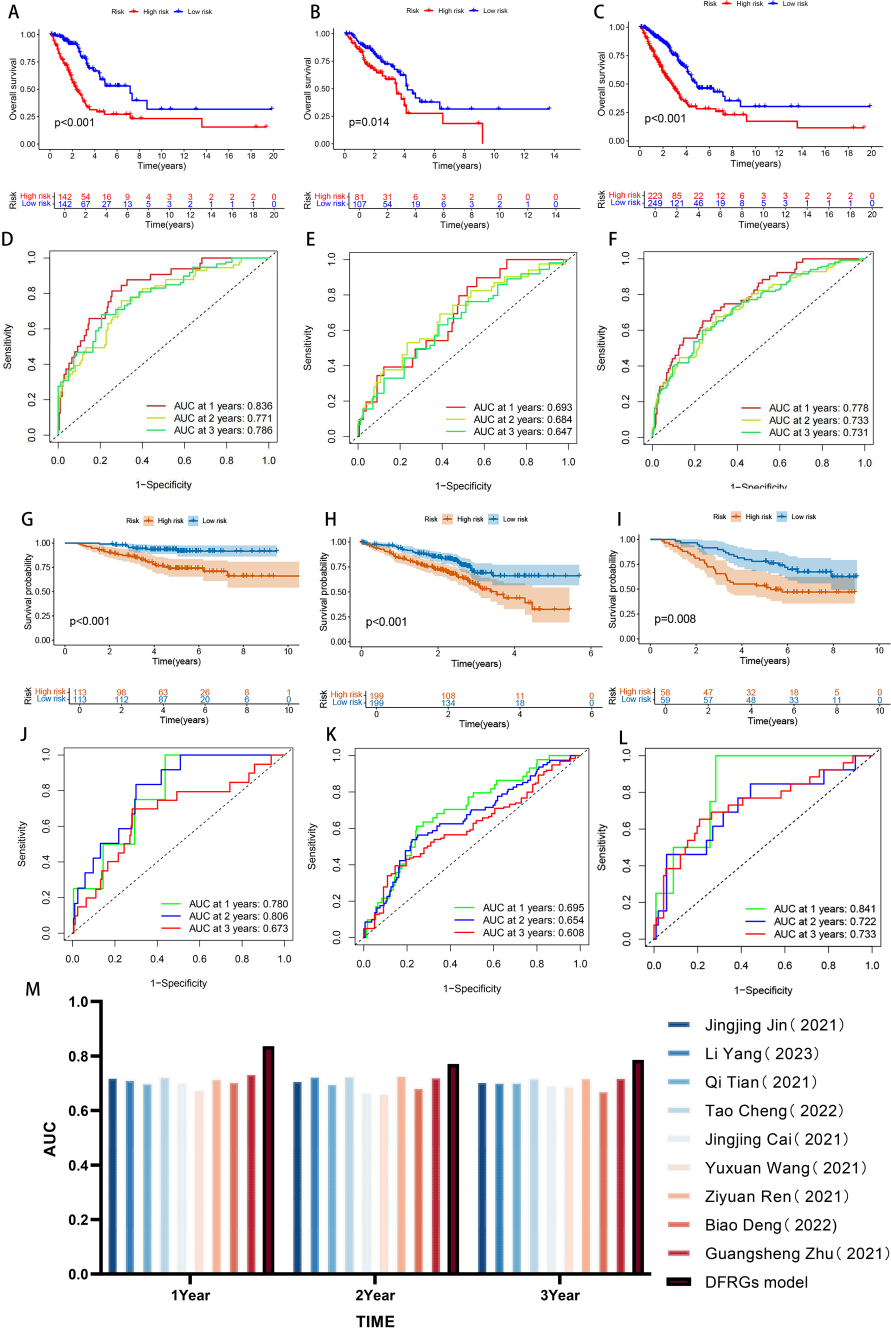
Evaluation and validation of prognostic risk models. **(A-C)** Kaplan-Meier survival curves in patients in the high-risk and low-risk groups were assessed in the Training set, internal validation set, and total set. **(D-F)** ROC curve analysis of training set, internal validation set, and total set. **(G-I)** Kaplan-Meier survival curves in patients in the high-risk and low-risk groups were assessed in GSE30210,GSE72094 and GSE13213 data sets. **(J-L)** ROC curve analysis of GSE30210, GSE72094 and GSE13213 data sets. **(M)** Bar chart comparing AUC with other authors.

By evaluating the expression levels of the six DFRGs in LUAD patients, we calculated each patient’s risk score using the risk score formula, divided the 472 LUAD patients into a training cohort with a 6:4 ratio, and divided all LUAD patients into high and low risk groups based on median patient risk score. The K-M survival curve analysis showed significant differences in OS between both high and low risk groups in the training set, internal validation set and total set (all *P* < 0.001, **Figures 3A–C**). The OS in the low risk group was significantly higher than that in the high risk group, suggesting the prognostic value of risk score for LUAD patients. ROC curves were plotted using the “timeROC package”. The results showed that the AUC values of the model for 1-year, 2-year, and 3-year survival rates in the training set were 0.836, 0.771, and 0.786, respectively, while those in the validation set were 0.693, 0.684, and 0.647, respectively. These findings indicate that the constructed model has good predictive performance (**Figures 3D–F**).

To further confirm the accuracy of the model, we performed the validation in three independent GEO external validation cohorts. The K-M survival curve analysis showed that the OS of the high and low risk groups was significantly different in both validation sets (all *P* < 0.001, **Figures 3G–I**), and the OS in the low risk patients was significantly higher than that in the high risk group, and the results were consistent with the TCGA database, suggesting the prognostic value of the risk score for LUAD patients. The ROC curve shows that the AUC of the 1, 2, and 3 years survival rates of GSE30210 were 0.780, 0.806, and 0.673, respectively. The AUC of the 1, 2, and 3 years survival rates of GSE72094 were 0.695, 0.654, and 0.608, respectively. The AUC of the 1, 2, and 3 years survival rates of GSE13213 were 0.841, 0.722, and 0.733, respectively. The expression results of prognosis-related DFRGs in the validation sets were consistent with those in the TCGA training cohort, indicating that the model we constructed had good predictive performance and could more accurately predict the prognosis of patients (**Figures 3J–L**). Nine previous models were compared through literature search (24–32), and the results revealed the highest AUC in 1, 2, and 3 years, which further demonstrated the predictive power of the model (**Figure 3M**). Therefore, comprehensive internal verification, external verification and literature review further verified the predictive ability of the disulfate-related ferroptosis lung adenocarcinoma prognosis model constructed in this study, and also revealed its important significance in the prognosis of lung adenocarcinoma.

### 3.4 Construction and evaluation of the prognostic nomogram

**Figure 4:**
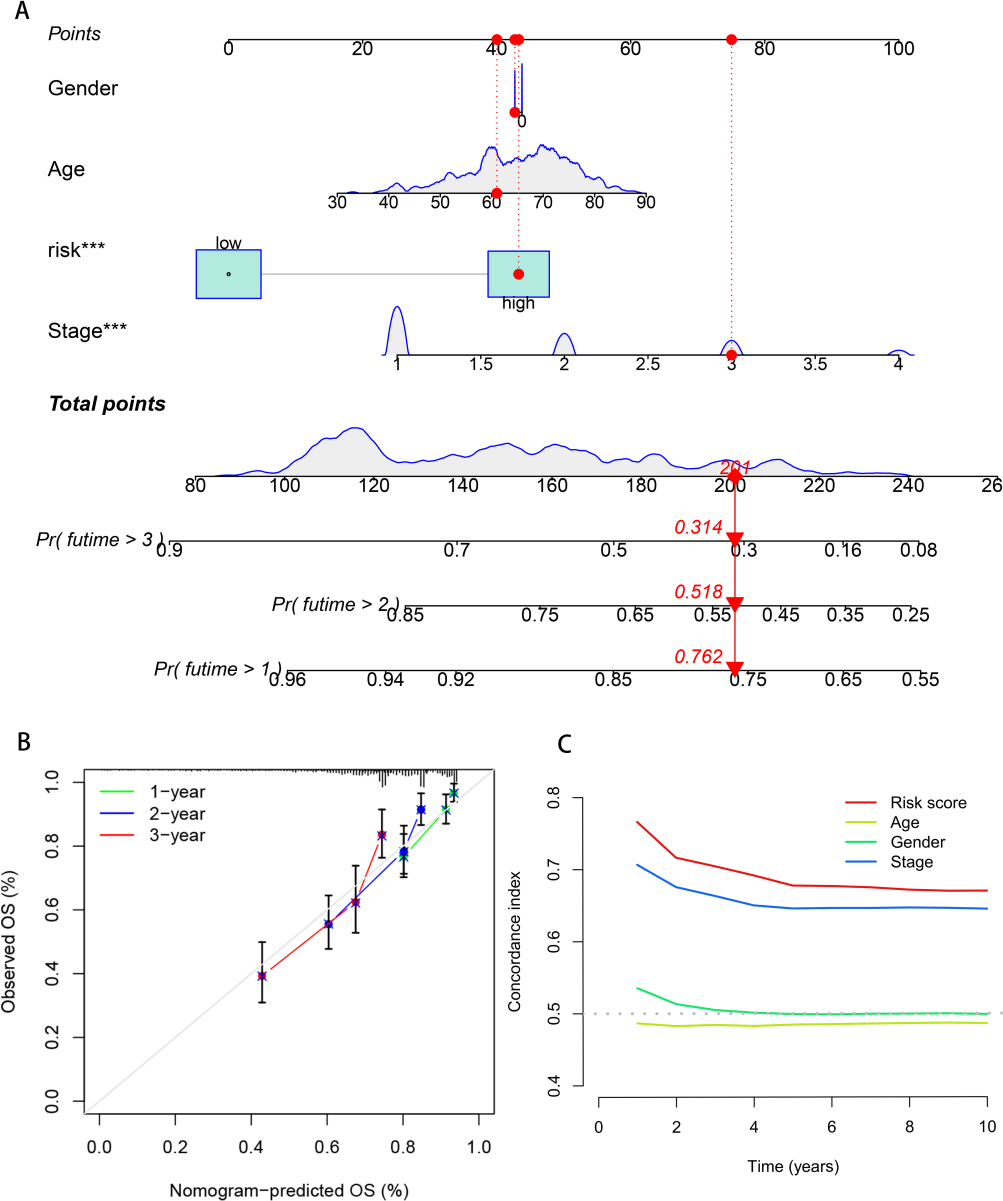
Construction and evaluation of the prognostic model in the nomogram. **(A)** Nomogram model of breast cancer patients. **(B)** The calibration curve of the nomogram predicts 1, 2, and 3 years survival of breast cancer patients. **(C)** Clinical efficacy of the DCA evaluation model.

To quantify the individual risk assessment of LUAD patients, this study integrated clinical characteristics (age, gender, stage) with the risk score to construct a nomogram model, and the prognosis was presented based on the scores of each prognostic factor. A patient was randomly selected for prediction. The model showed that the total score of this patient was 201 points, and the 1, 2, and 3 years survival rates after the diagnosis of LUAD were 76.2%, 51.8% and 31.4%, respectively, as shown in **Figure 4A**. The calibration curve indicated that the 1, 2, and 3 years survival rates predicted by the nomogram for LUAD patients were in good agreement with the actual values, as shown in **Figure 4B**. The index of concordance (C-index) evaluates the degree of conformity between the model’s predicted results and the actual observed results, thereby assessing the predictive accuracy of the model. The curve of the combined model with clinical pathological characteristics was higher than that of a single clinical characteristic, indicating that the combined model can enhance the accuracy of predicting the prognosis of LUAD patients (**Figure 4C**). Based on the above results, it can be concluded that the prediction model in this study has a robust and accurate ability to predict the prognosis and can provide a reference for clinical management and decision-making.

### 3.5 Immune-related analysis of the prognostic model of DFRGs in LUAD

**Figure 5:**
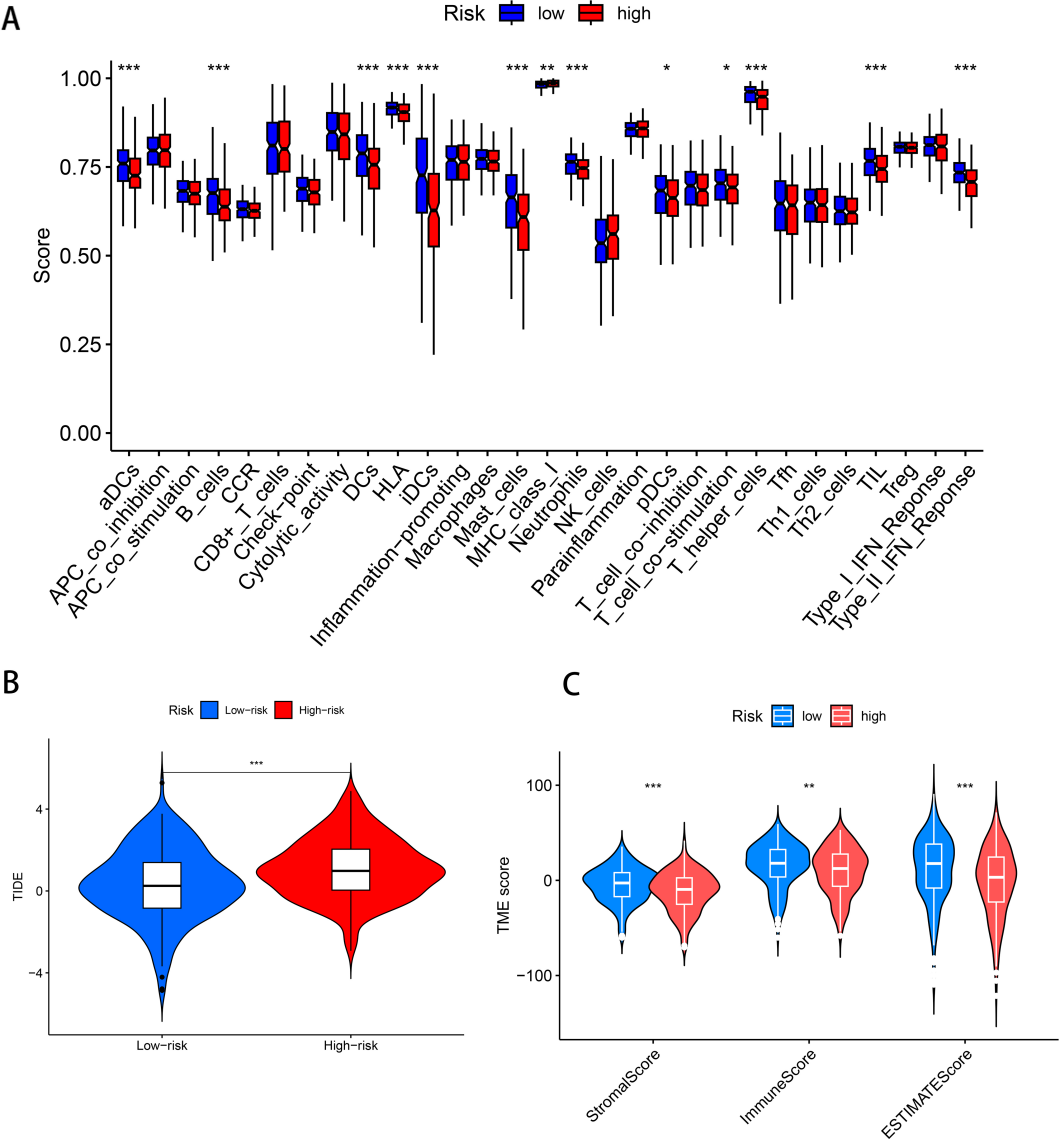
Immune-related analysis of the prognostic model of DFRGs in LUAD. **(A)** Immune-related functional differences analysis. **(B)** TIDE scores in patients in the high-risk and low-risk groups. **(C)** Analysis of the differences in stromal cells, immune cells and comprehensive scores between patients in the high-risk and low-risk groups. (***P < 0.001, **P < 0.01, *P < 0.05).

The ssGSEA was used to investigate the differences in immune functions between the two groups. The results showed that among the two groups with statistically significant differences, immune functions were more enriched in the low-risk group, such as B cells (*P* < 0.001), aDCs (*P* < 0.001), DCs (*P* < 0.001), iDCs (*P* < 0.001), Type II IFN Response (*P* < 0.001), etc., indicating that the immune function in the low-risk group was more active (**Figure 5A**). The TIDE score represents the probability of immune escape and further indicates the likelihood of resistance to immunotherapy. The results showed that the probability of immune escape was higher in the high-risk group (*P* < 0.01, **Figure 5B**), suggesting that patients in the high-risk group were less likely to benefit from immunotherapy and that the effect of immunotherapy might be better in patients in the low-risk group. To explore the infiltration of immune cells between the two groups, a TME difference analysis was performed, which showed that the differences in stromal cells, immune cells, and comprehensive scores between the high-risk and low-risk groups were all statistically significant and that the scores in the low-risk group were higher in all three groups (*P* < 0.005, **Figure 5C**). Therefore, comprehensive internal validation, external validation, and comparative literature research further confirm the predictive capability of the disulfidptosis-ferroptosis-related prognostic model for lung adenocarcinoma constructed in this study, and also reveal its significant implications in the prognosis of lung adenocarcinoma.

### 3.6 TMB analysis

**Figure 6:**
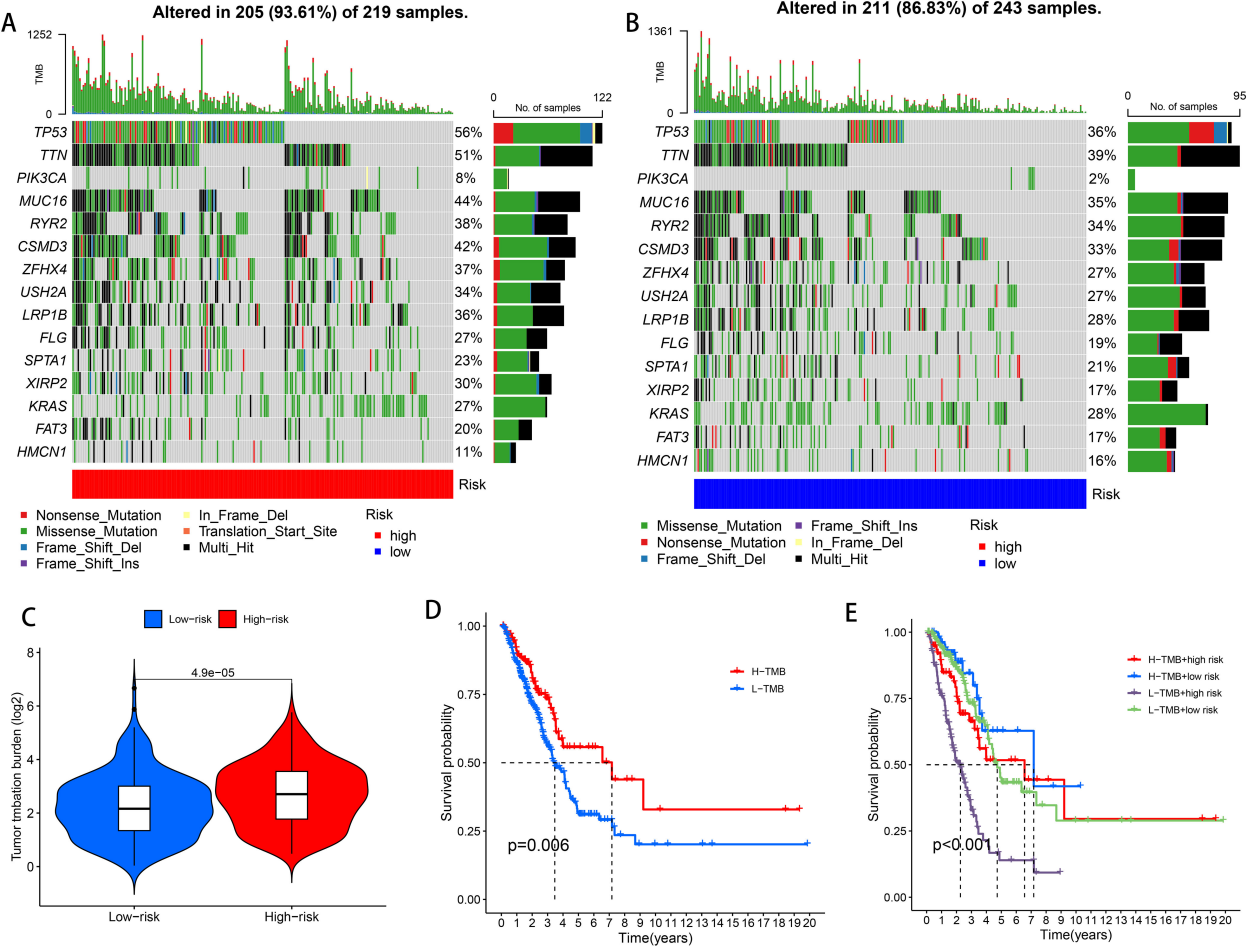
High-risk and low-risk groups of TMB. **(A)** TMB waterfall plot of the top 15 genes in the high risk group. **(B)** TMB waterfall plot of the top 15 genes in the low risk group. **(C)** Analysis of TMB differences in the high and low risk groups. **(D)** Kaplan-Meier survival analysis of LUAD patients in the high TMB and low TMB groups. **(E)** Kaplan-Meier survival analysis of TMB combined with risk score in LUAD patients.

Tumor Mutation Burden (TMB), which calculates the number of somatic mutations per megabase in a genomic sequence, is a potential predictive biomarker in many solid tumors critical for tumor prognosis and can be used to predict the effectiveness of immune checkpoint inhibitors on tumors. To investigate the differences in cancer-associated gene mutations between the high-risk and low-risk groups, we obtained somatic mutation data from TCGA-LUAD. Examination identified the 15 most frequently mutated genes, including TP 53, TTN, PIK3CA, MUC 16, RYR 2, CSMD 3, ZFHX 4, USH 2 A, LRP 1 B, FLG, SPTA 1, XIRP 2, KRAS, FAT 3, and HMCN 1 (**Figures 6A, B**). TMB difference analysis violin plots showed differences in TMB between the high and low risk groups (*P* < 0.001), and TMB was significantly higher in the high risk than the low risk group (**Figure 6C**). According to the mutation status, we divided the patients into high TMB and low TMB groups. High TMB can a survival benefit for LUAD patients, as shown in **Figure 6D** in the high TMB group than in the low TMB group (*P* = 0.006). Next, a group analysis combining TMB and risk score showed that HTMB + low-risk patients had the best prognosis (*P* < 0.001), and their seven-year survival rate was about 50%. In contrast, patients with a high risk of LTMB + had the worst prognosis, with a 7-year survival rate of less than 20% (**Figure 6E**).

### 3.7 Single-cell sequencing analysis

**Figure 7:**
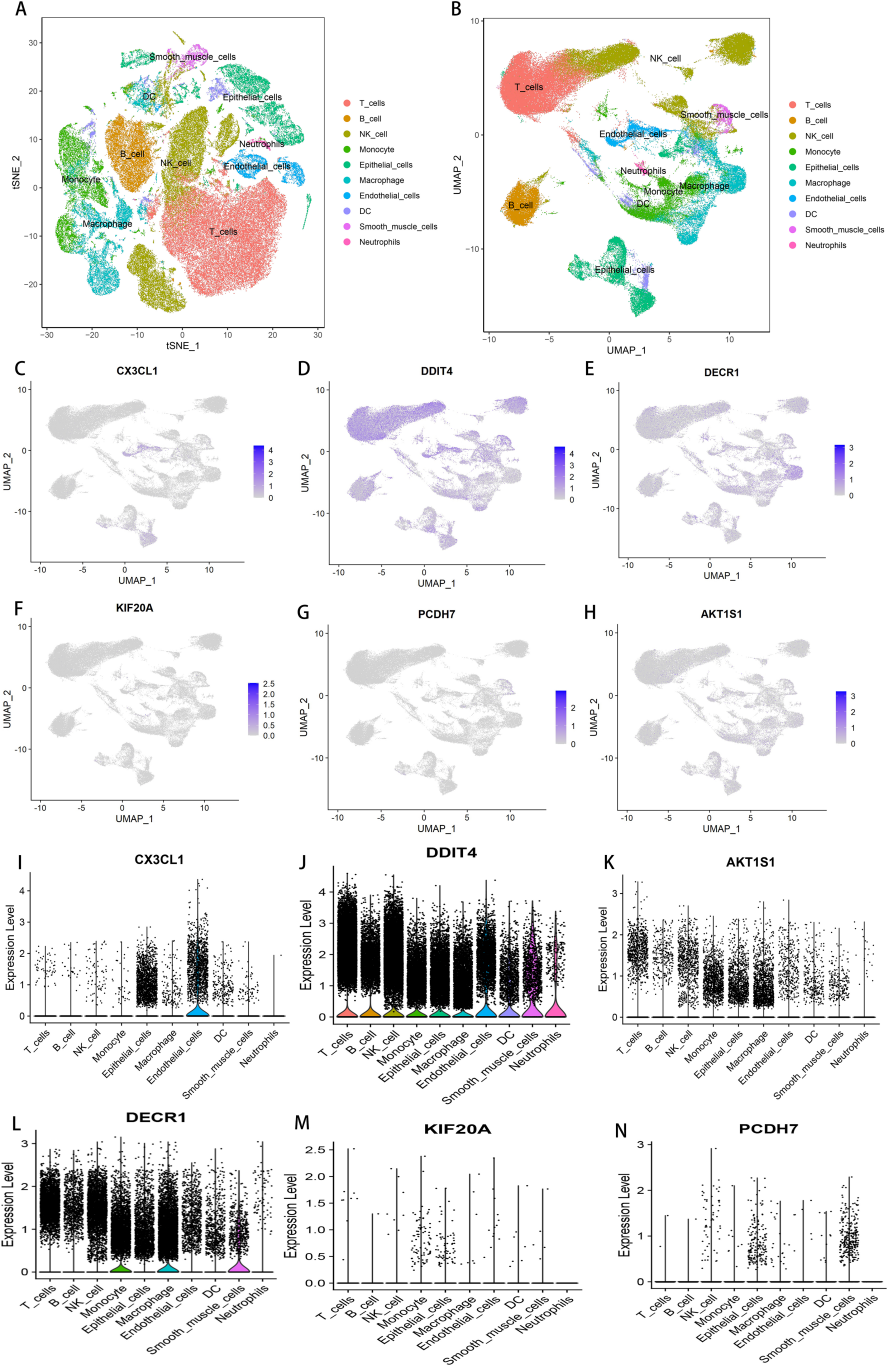
Single-cell sequencing and analysis. **(A)** t-SNE cluster annotation diagram. **(B)** UMAP cluster annotation map. **(C)** Cellular expression level of CX3CL1 in GSE189357. **(D)** Cellular expression level of DDIT4 in GSE189357. **(E)** Cell expression level of KIF20A in GSE189357. **(F)** Cellular expression level of PCDH7 in GSE189357. **(G)** Cellular expression level of DECR1 in GSE189357. **(H)** Cellular expression level of AKT1S1 in GSE189357. **(I)** Cellular expression level of CX3CL1 in GSE189357. **(J)** Cellular expression level of DDIT4 in GSE189357. **(K)** Cellular expression level of AKT1S1 in GSE189357. **(L)** Cellular expression level of DECR1 in GSE189357. **(M)** Cell expression level of KIF20A in GSE189357. **(N)** Cellular expression level of PCDH7 in GSE189357.

In this study, nine samples from GSE189357 were used for single-cell sequencing analysis, annotated by t-SNE and UMAP clustering: 10 cell types including T cells, B cells, natural killer cells, monocytes, epithelial cells, macrophages, endothelial cells (**Figures 7A, B**). Cell expression levels were separately for the six DFRGs in the model using UMAP clustering, The results showed that DDIT 4 expression was higher in all of the 10 cell types. The expression levels of AKT1S1, KIF20A and PCDH 7 were relatively low in 10 cell types. DECR1 is relatively highly expressed in monocytes, macrophages, and smooth muscle cells. CX3CL1 Is relatively high expression in epithelial cells (**Figures 7C–N**). These results indicate that the six model genes are clustered in single cells of lung adenocarcinoma, which provides a basis for subsequent biological molecular research.

### 3.8 Drug sensitivity analysis

**Figure 8:**
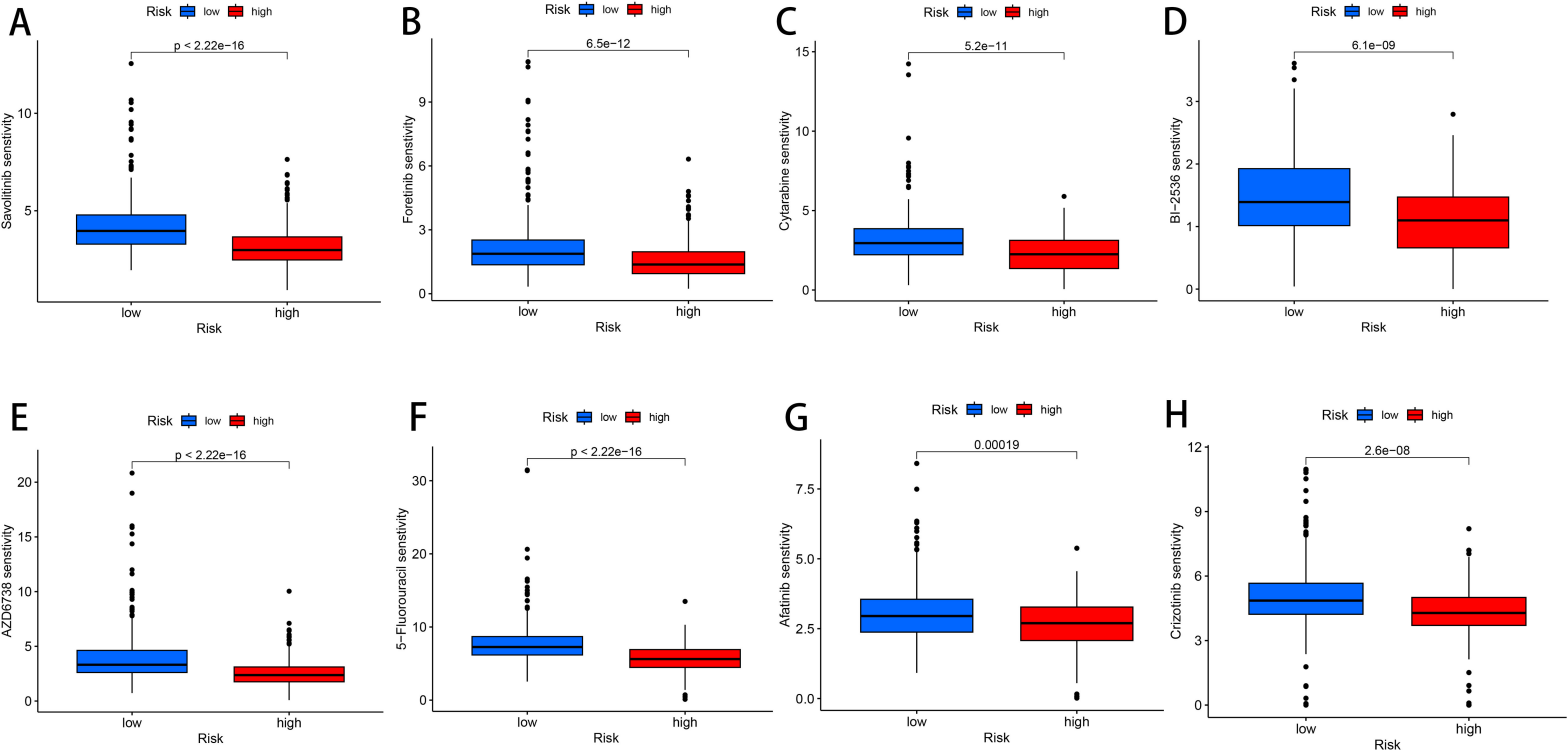
Drug susceptibility analysis. **(A)** Savolitinib. **(B)** Foretinib. **(C)** Cytarabine. **(D)** BI-2536. **(E)** AZD6738. **(F)** 5-Fluorouracil. **(G)** Afatinib. **(H)** Crizotinib.

To search for drugs potentially effective for the treatment of LUAD, predicting differences in chemotherapeutic drug sensitivity in patients with different risk groups, We performed a Wilcoxon-test to compare the differences between the two risk groups, LUAD patients, Where the abscissa represents patients in the LUAD high and low risk group, The ordinate represents the IC50-value, We found lower IC50 values in the high-risk group of patients with voritinib, foritinib, cytarabine, PLK inhibitors, selective ATR kinase inhibitors, 5-fluorouracil, afatinib, tyrosine kinase receptor inhibitors, It indicates that the patients in the high risk group had higher sensitivity to 8 drugs, foritinib, cytarabine, PLK inhibitor, selective ATR kinase inhibitor, 5-fluorouracil, afatinib, tyrosine kinase receptor inhibitor, More favorable to receiving this drug (*P* < 0.01, **Figures 8A–H**). Therefore, the high and low risk groups distinguished by the risk score of this prognostic model were different in drug sensitivity, indicating that this prognostic model has some significance in drug use.

### 3.9 Detection of DECR1 function in lung adenocarcinoma cells

**Figure 9:**
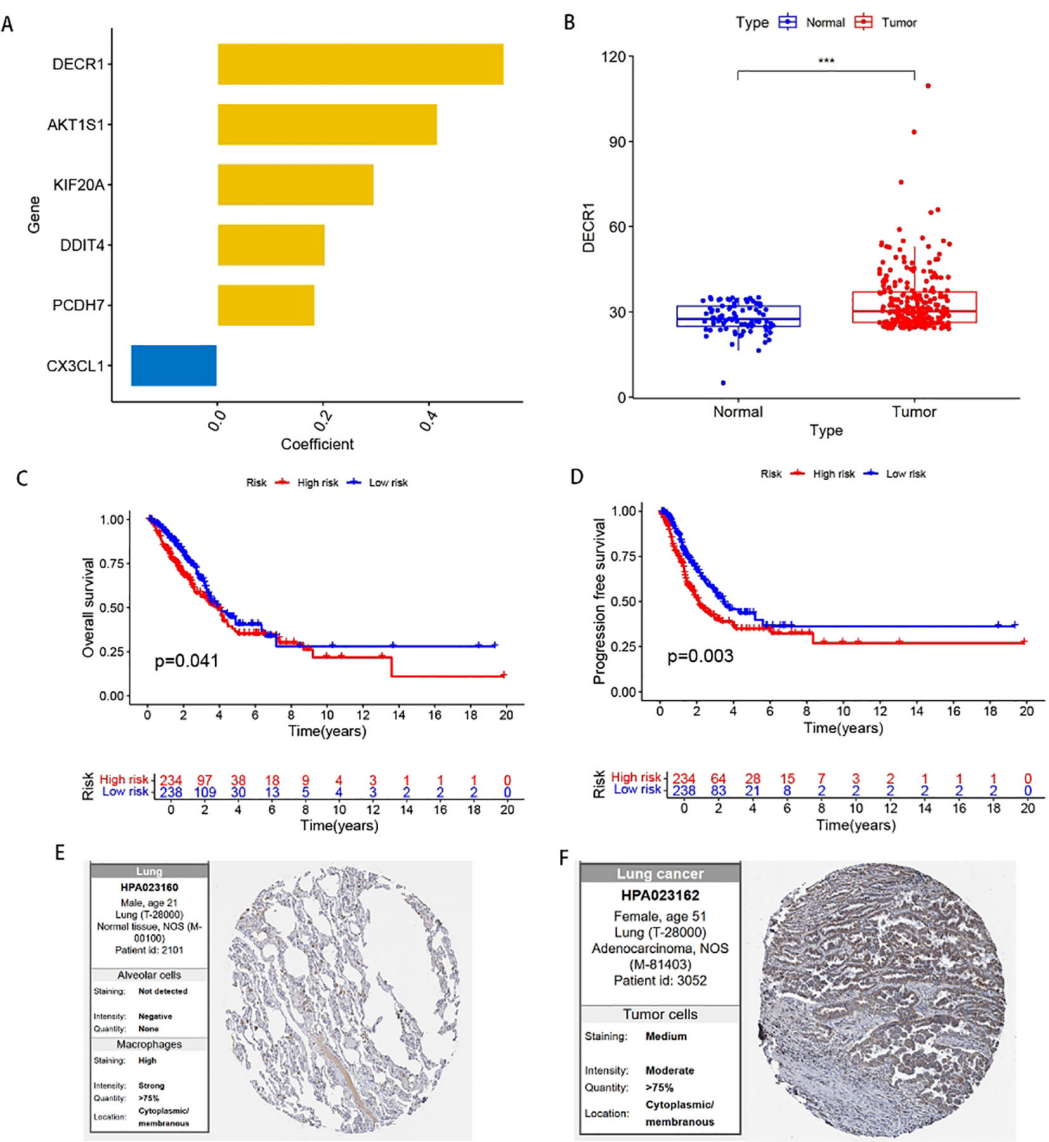
Expression of DECR1 in LUAD. **(A)** Ranking of the importance of six FFRGs in the prognostic model of TCGA-LUAD-DFRGs. **(B)** Differential expression of DECR1 in normal and tumor samples. **(C)** K-M curve of OS. **(D)** K-M curves of PFS. **(E)** Protein expression of DECR1 in normal tissues in HPA database. **(F)** Protein expression of DECR1 in lung adenocarcinoma tissue in HPA database.

In order to find the key regulatory genes of disulfide death-related iron death in LUAD, this study first analyzed the six DFRGs that constructed the model based on the TCGA database and ranked the importance, and showed that the DECR1 importance coefficient was 0.542 (**Figure 9A**). Subsequently, the differential analysis of DECR1 expression in normal and tumors in the TCGA-LUAD database showed that the expression of DECR1 was higher in tumor samples than in normal samples (**Figure 9B**). Thereafter, the K-M survival analysis of DECR1 in the TCGA-LUAD risk outcome model and progression-free survival (PFS) in LUAD-DFRGs was statistically significant in the high and low risk groups (**Figures 9C, D**). Meanwhile, the protein expression level of DECR 1 in lung adenocarcinoma tissues was also significantly higher than that in normal tissues (**Figures 9E, F**). Therefore, based on the above results, DECR1 was selected as the key DFRGs in lung adenocarcinoma for further subsequent expression and biological function studies.

**Figure 10:**
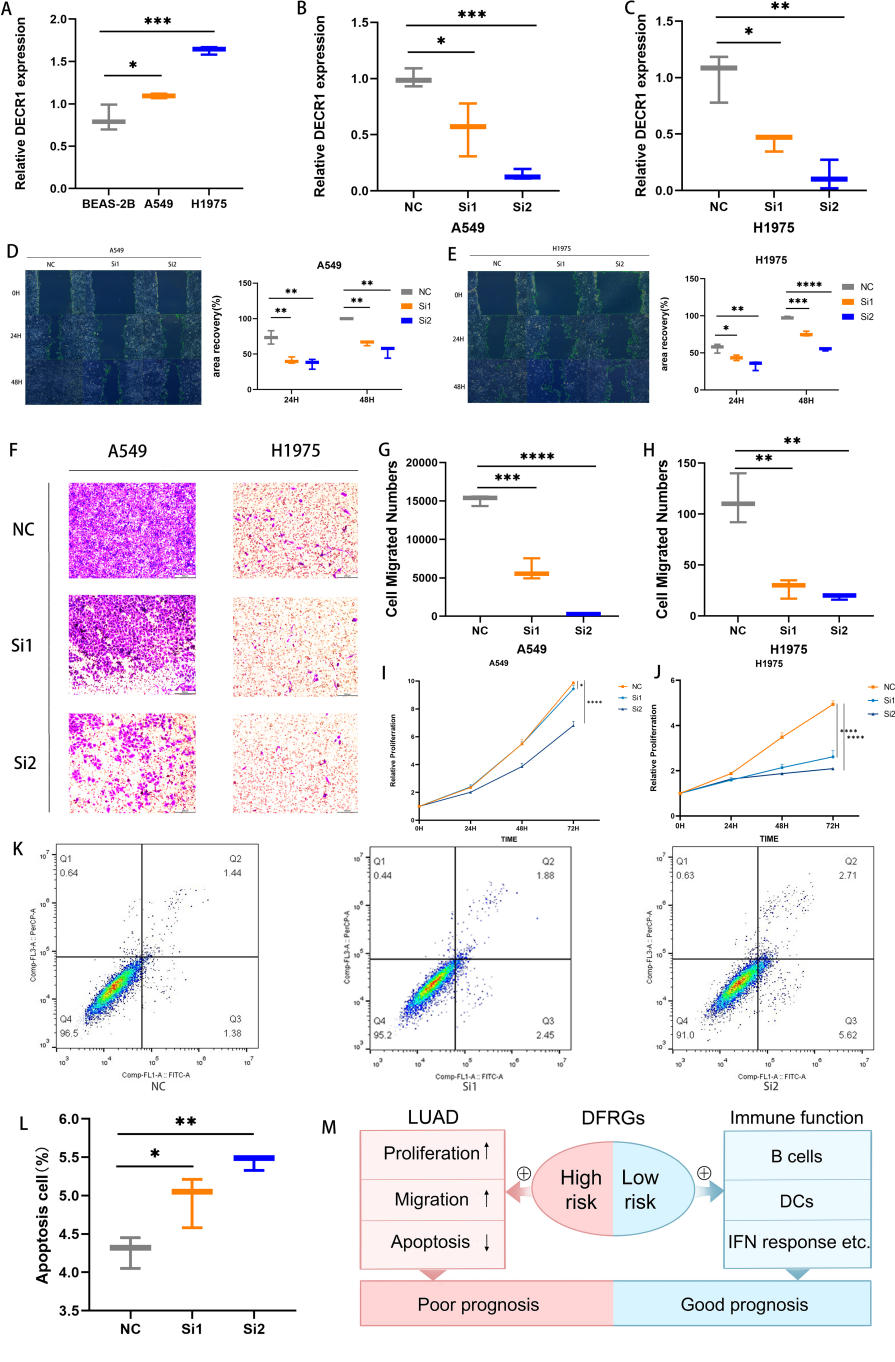
Detection of DECR1 function in lung adenocarcinoma cells. **(A)** The expression level of DECR1 in BEAS-2B, A549, and H1975 was determined by qRT-PCR. **(B, C)** The expression level of knockdown DECR1 in A549 and H1975 was measured by qRT-PCR. **(D, E)** The migration of knockdown DECR1 in A549 and H1975. **(F-H)** Transwell experimental test for the migration of knockdown DECR1 in A549 and H1975. **(I, J)** CCK 8 test for the proliferation of knockdown DECR1 in A549 and H1975. **(K, L)** Apoptosis of knockdown DECR1 in A549. **(M)** Mechanism diagram of the prognostic role of DFRGs in LUAD.

qRT-PCR was used to evaluate the relative expression levels of DECR1 in human normal lung epithelial cells BEAS-2B and lung adenocarcinoma cell lines A549 and H1975 (**Figure 10A**). Compared with lung adenocarcinoma cell lines A549 and H1975, the expression of DECR1 in human normal lung epithelial cells BEAS-2B was significantly lower. Two kinds of siRNAs were transfected into two different cell lines to investigate the *in vitro* regulatory role of DECR1 on LUAD. qRT-PCR experiments showed that the expression levels of Si-DECR1–1 and Si-DECR1-2 (subsequently referred to as Si1 and Si2) were significantly lower than that of the negative control (NC), indicating that the knockdown was successful (**Figures 10B, C**). The results of the wound healing experiment showed that the migration speeds of Si1 and Si2 in A549 and H1975 cell lines were significantly slower than that of NC (**Figures 10D, E**). In the transwell experiment, the number of migrating cells of Si1 and Si2 in A549 and H1975 cell lines was less than that of Si-NC (**Figures 10F–H**). These results suggest that knocking down DECR1 can inhibit the migratory ability of lung adenocarcinoma cells. The CCK-8 experiment demonstrated that knocking down DECR1 inhibited the proliferation of A549 and H1975 (**Figures 10I, J**). In addition, the apoptosis experiment indicated that knocking down DECR1 increased the apoptosis of lung adenocarcinoma cell A549 and inhibited cell survival (**Figures 10K, L**). Therefore, according to the results of migration, apoptosis and proliferation of DECR1 in lung adenocarcinoma cells, it is suggested that DECR1 may promote the development of lung adenocarcinoma and may be an effective prognostic molecular marker for lung adenocarcinoma, which can provide a basis for further mechanism research.

## 4 Discussion

Cell death is emerging as a new focus in tumor therapy. Previous studies have demonstrated that ferroptosis-related genes can provide certain insights for improving the prognosis of patients with lung adenocarcinoma. And disulfidptosis, as a newly discovered form of cell death, has been proven to play a crucial role in tumor progression and cancer treatment (33). Therefore, in this study, we combined ferroptosis genes with disulfidptosis genes to construct a prognostic model for LUAD patients based on DFRGs, aiming to identify novel prognostic biomarkers. Furthermore, we analyzed the clinical values of TIME, TMB, and drug sensitivity in LUAD patients. Additionally, molecular biology experiments were conducted to investigate the functions of potential biomarkers in cell migration, proliferation, and apoptosis in LUAD. This study is expected to provide new insights for the personalized clinical treatment of LUAD patients.

Based on the TCGA public database, this study established a prognostic model for LUAD that includes 6 DFRGs, namely AKT1S1, DDIT4, DECR1, KIF20A, PCDH7, and CX3CL1. Based on the above 6 DFRGs, patients are categorized into high-risk and low-risk groups. The high-risk group promotes the proliferation and migration of lung adenocarcinoma cells while inducing the apoptosis of these cells, and the combined effects of these factors result in a poor prognosis for patients. The low-risk group exhibits stronger immune function, marked by the presence and activity of key immune cells such as B cells, DCs, leading to better patient outcomes (**Figure 10M**). For the DFRGs risk model constructed in this study, the AUC values for predicting 1-year, 2-year, and 3-year survival rates were 0.836, 0.771, and 0.786 in the training set; 0.693, 0.684, and 0.647 in the validation set; and 0.778, 0.733, and 0.731 in the combined dataset. All these values are superior to those of the previously published ferroptosis-gene-based models, which proves that the DFRG prognostic model established in this study has better accuracy and reliability. In the external validation, the ROC results of datasets GSE30210 (1-year, 2-year, 3-year AUC = 0.780, 0.806, 0.673), GSE72094 (0.695, 0.654, 0.608), and GSE13213 (0.841, 0.722, 0.733) further confirmed the good extrapolability of the model.

Previous studies have found that AKT1S1 is a direct target of miR-30c-2-3p in gastric cancer cells. Moreover, AKT1S1 is upregulated in hepatocellular carcinoma and is associated with the poor prognosis of patients with hepatocellular carcinoma and promotes the growth of hepatocellular carcinoma (34, 35). DDIT4 participates in the regulation of autophagy in triple-negative breast cancer. Knocking down DDIT4 significantly inhibits the tumor progression of triple-negative breast cancer both *in vitro* and *in vivo*, and inhibiting DDIT4 can enhance the efficacy of paclitaxel in patients with triple-negative breast cancer (36). KIF20A is a member of the kinesin family. It transports chromosomes during mitosis and plays a key role in cell division. It is highly expressed in multiple cancers, participates in cancer progression by regulating cell division, and is related to drug or chemotherapy resistance in tumor treatment (37). PCDH7, known as protocadherin 7, is a subfamily of the cadherin superfamily and plays biological roles in multiple cancer types. It significantly promotes the development of lung cancer and is related to cisplatin resistance, and it is expected to become a potential therapeutic target (38). An increase in the expression of CX3CL1 will lead to an increase in the anti-tumor immune response, thereby reducing the rate of tumor growth and improving the survival rate of experimental animals and cancer patients. Increasing the expression of CX3CL1 in tumors has a therapeutic effect and can be used as one of the elements of immunotherapy and as an auxiliary means to improve the efficacy of anti-cancer treatment (39).

In this study, the nomogram integrating LUAD prognosis model risk scores and clinical features demonstrated robust stability and accuracy in survival prediction, providing a novel clinical tool for prognostic assessment (40). Based on the DFRGs model risk stratification, LUAD patients were categorized into high- and low-risk groups. Immunological correlation analysis revealed that the low-risk group exhibited elevated levels of B cells, CD4+ T cells, and CD8+ T cells compared to the high-risk cohort (41). Furthermore, application of the TIDE algorithm to evaluate immunotherapy potential demonstrated that high-risk patients had significantly higher TIDE scores and lower effective immunotherapy response rates, suggesting that this subgroup may derive greater benefit from immunotherapy, thereby offering new perspectives for LUAD clinical management (42). Our findings on the distinct immune characteristics between different risk groups resonate with comprehensive pan-cancer analyses of metabolic cell death pathways (43), further validating the biological plausibility of our model. Additionally, we investigated the correlation between risk scores and sensitivity to eight common therapeutic agents, including chemotherapy and targeted drugs to assess the model’s translational value (44). The analysis demonstrated that high-risk patients exhibited significantly higher sensitivity to eight specific drugs, including 5-fluorouracil and afatinib. This enhanced drug responsiveness implies that targeted administration of these agents to high-risk patients could achieve more pronounced tumor suppression and improved therapeutic outcomes (45), establishing a solid theoretical foundation for personalized drug selection in LUAD treatment.

DECR1 is a mitochondrial enzyme that participates in the metabolism and beta-oxidation of unsaturated fatty acid alpha-keto A esters and is located on chromosome 8q21.3. It plays a role in redox balance by regulating the ratio of saturated phospholipids to unsaturated phospholipids. Polyunsaturated fatty acids (PUFA) accumulate within cells, eventually leading to lipid peroxidation and iron deficiency. When DECR1 is deleted, castration-resistant prostate cancer cell lines are more likely to undergo ferroptosis due to endoplasmic reticulum stress induction (46, 47). Moreover, DECR1 is closely related to lipid metabolism, ferroptosis, mitochondria and tumorigenesis. This is the first time DECR1 has been associated with LUAD. The research results indicated that knocking down DECR1 can restrain the migration and proliferation abilities of lung adenocarcinoma cells and simultaneously accelerate their apoptosis, which is consistent with the results of the model. It is highly likely that DECR1 promotes the occurrence and development of lung adenocarcinoma and can be further studied as an effective biomarker.

In conclusion, we screened the DFRGs and established and validated a prognostic model on this basis. This model can well predict the OS of LUAD patients and conduct biological verification on the key genes. However, there are still certain deficiencies in this study. First, the core analysis of this study is based on the TCGA dataset, and relevant studies have confirmed its value in mining molecular mechanisms and screening biomarkers (48). But bulk transcriptome data inherently suffer from cohort selection bias, heterogeneous clinical annotations, and platform artifacts (49, 50), which may affect the reliability of DFRGs screening and model construction. Additionally, this study only carried out *in vitro* functional validation on DECR1. The biological functions of the other five genes have not been confirmed through experiments, and most of the work relied on the secondary analysis of public databases, lacking more in-depth exploration of molecular mechanisms and experimental validation, both of which are crucial for confirming the translational application value of the biomarkers (51). Therefore, further work is needed in the follow-up to verify the accuracy of the model.

## Data Availability

The datasets presented in this study can be found in online repositories. The names of the repository/repositories and accession number(s) can be found in the article/Supplementary Material.
